# Targeting mTOR as a Therapeutic Approach in Medulloblastoma

**DOI:** 10.3390/ijms19071838

**Published:** 2018-06-22

**Authors:** Juncal Aldaregia, Ainitze Odriozola, Ander Matheu, Idoia Garcia

**Affiliations:** 1 Cellular Oncology Group, Biodonostia Research Institute, 20014 Donostia-San Sebastián, Spain; juncal.aldareguia@biodonostia.org (J.A.); ainitzeodri15@gmail.com (A.O.); ander.matheu@biodonostia.org (A.M.); 2 IKERBASQUE, Basque Foundation for Science, 48013 Bilbao, Spain; 3 CIBER de fragilidad y envejecimiento saludable (CIBERfes), 28029 Madrid, Spain; 4 Physiology Department, Faculty of Medicine and Nursing, University of the Basque Country (UPV/EHU), 48940 Leioa, Spain

**Keywords:** mTOR, Medulloblastoma, MBSCs

## Abstract

Mechanistic target of rapamycin (mTOR) is a master signaling pathway that regulates organismal growth and homeostasis, because of its implication in protein and lipid synthesis, and in the control of the cell cycle and the cellular metabolism. Moreover, it is necessary in cerebellar development and stem cell pluripotency maintenance. Its deregulation has been implicated in the medulloblastoma and in medulloblastoma stem cells (MBSCs). Medulloblastoma is the most common malignant solid tumor in childhood. The current therapies have improved the overall survival but they carry serious side effects, such as permanent neurological sequelae and disability. Recent studies have given rise to a new molecular classification of the subgroups of medulloblastoma, specifying 12 different subtypes containing novel potential therapeutic targets. In this review we propose the targeting of mTOR, in combination with current therapies, as a promising novel therapeutic approach.

## 1. Mechanistic Target of Rapamycin (mTOR)

**Figure 1:**
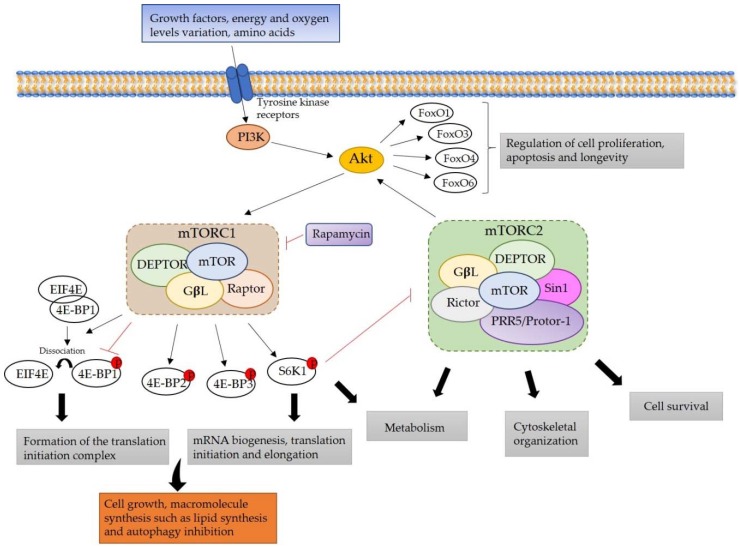
Mechanistic target of rapamycin (mTOR) signaling pathway. mTOR is part of two different complexes, mTOR complex 1 (mTORC1) and complex 2 (mTORC2). mTORC1 is activated by the phosphatidylinositol 3-kinase (PI3K)/AKT signaling pathway, and its downstream effectors activate cell growth, lipid synthesis, and metabolism, whereas it inhibits the autophagy. This complex can be inhibited by rapamycin. mTORC2 activates AKT, thus activating also mTORC1. Furthermore, mTORC2 activates the metabolism, cytoskeletal organization, and cell survival.

The mechanistic (formerly mammalian) target of rapamycin (mTOR) is a master signaling pathway that regulates organismal growth and homeostasis. This pathway is not only implicated in physiological conditions but it is also central in several pathological conditions [1]. mTOR is highly sensitive to rapamycin, a specific inhibitor of this serine/threonine kinase and an antiproliferative drug that is used clinically in antitumor and immunosuppressive therapy [2]. As represented in Figure 1, mTOR is activated by tyrosine kinase receptors via the phosphatidylinositol 3-kinase (PI3K)/AKT pathway. mTOR interacts with different proteins, forming two functionally distinct multiprotein complexes, mTOR complex 1 (mTORC1) and complex 2 (mTORC2) [3]. mTORC1 is composed of mTOR, Raptor, GβL, and DEP domain-containing mTOR-interacting protein (DEPTOR) [4]. It is a sensor of a wide variety of cellular signals, including growth factors, energy levels, oxygen, stress, or amino acids. These signals promote the regulation of cell growth and metabolism through a number of downstream effects, such as protein and lipid synthesis or autophagy inhibition [3]. Less is known about mTORC2, which is composed of mTOR, Rictor, GβL, Sin1, Proline rich protein 5 (PRR5)/Protor-1, and DEPTOR [5]. It responds to growth factors that control the cell proliferation, but it is insensitive to nutrients. mTORC2 can directly phosphorylate AKT, and it controls the cytoskeletal organization and cell survival [6] (Figure 1).

One of the most important downstream effects of mTORC1 is the upregulation of protein synthesis. It phosphorylates several translation regulators, including eukaryotic translation initiation factor 4E (eIF4E)-binding proteins (4E-BP1, 2, 3) [1] and the p70 ribosomal S6 kinase 1 and 2 (S6K1) [7]. The phosphorylation of 4EBP1 inactivates this protein and allows the dissociation of 4EBP1 from EIF4E, enabling the formation of the translation initiation complex. In the case of S6K1, when phosphorylated, it increases mRNA biogenesis, translational initiation, and elongation [1]. Furthermore, S6K1 establishes a negative-feedback mechanism between mTORC1 and mTORC2; when there is a strong activation of mTORC1, mTORC2 is inhibited [8,9]. Since mTORC2 activates AKT, the activation of mTORC1 indirectly inhibits AKT. AKT activation via mTORC2 is required for the phosphorylation of some AKT substrates, such as the members of the Forkhead boxO (FoxO) family (FoxO1, 3, 4, and 6), which are involved in the regulation of cellular processes such as cell proliferation, apoptosis, and longevity [9] (Figure 1). 

It is more and more obvious that the mTOR signaling pathway has an important role in essential cellular functions. The key role of mTOR in controlling cell proliferation has raised the interest in rapamycin for cancer therapy. This drug disrupts mTORC1 protein complex formation, whereas mTORC2 is quite insensitive to the drug, and long-term treatments are required in order to inhibit its assembly [10] (Figure 1).

## 2. mTOR in Central Nervous System (CNS) Development

mTOR plays an important role in the development of an organism because of its implication in the growth, proliferation, and migration of every cell during normal brain development [11]. Accordingly, the first mTOR knockout (KO) mouse study demonstrated that mTOR is indispensable for normal development and viability [11,12]. Indeed, the importance of mTOR in the brain development was evidenced when Hentges and colleagues created a loss of function mutant of mTOR and it showed a defect in the telencephalon formation, and it died in mid-gestation [13].

Nowadays, it is known that the deregulation of mTOR signaling is associated with many brain diseases, including neurological diseases, psychiatric diseases, or pediatric brain tumors [9,14]. The activation of the PI3K/AKT/mTOR signaling pathway enhances the proliferation of progenitors, neuronal hypertrophy, and excessive dendritic branching, while the opposite consequences are presented when the pathway is suppressed [15].

When forming the cerebellum, cerebellar granule neuron precursors (CGNPs) undergo a rapid expansion in the external granule layer (EGL) on the dorsal surface of the cerebellum. Afterwards, they exit the cell cycle, migrate internally, and differentiate into interneurons [16]. In the expansion phase, the CGNPs require Sonic Hedgehog (SHH) signaling activation for cell proliferation and insulin-like growth factor (IGF), which positively regulates the mTOR pathway, for cell survival capacity [16]. Furthermore, Mainwaring and Kenney demonstrated that SHH signaling modulates the individual mTOR effectors separately, in order to maintain a proliferation-competent state. Unlike what has been observed in cell lines, the CGNPs positively regulate eIF4E and negatively regulate S6K, promoting cell proliferation and cell cycle progression [16].

## 3. Medulloblastoma

**Figure 2:**
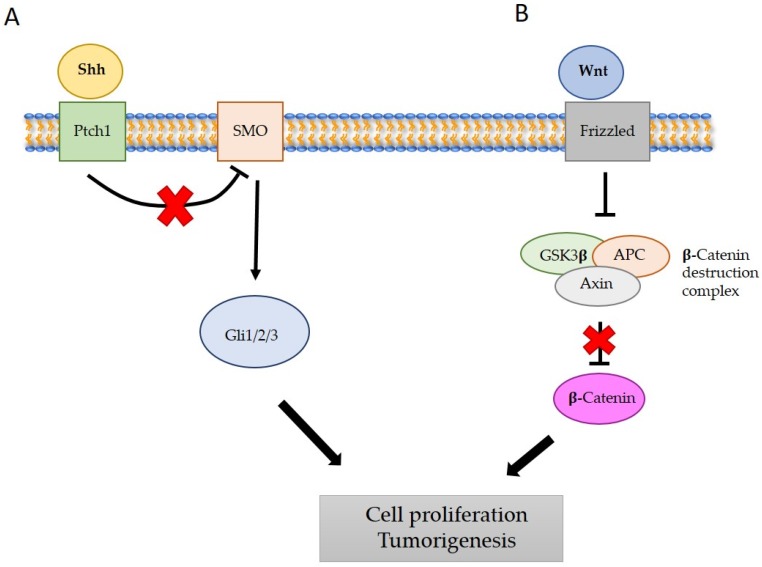
The Sonic Hedgehog (SHH)/Patched (PTCH1) and WNT signaling pathways. (**A**) The SHH ligand inactivates the PTCH1 receptor allowing Smoothened (SMO) to become active. Red cross represents the release of the inhibition exerted by PTCH1 on SMO when SHH is present. The SMO activates the GLI proteins, a family of transcription factors that turn on the expression of different target genes, giving raise to cell proliferation and tumorigenesis (activation of the pathway represented with black arrows). (**B**) The binding of WNT to the Frizzled receptor activates a cascade of downstream events, resulting in the inactivation of the β-catenin destruction complex. The red cross represent the release of the inhibition exerted by the β-catenin destruction complex on β-catenin. In consequence, β-catenin activates and promotes the transcription of genes that promote cell proliferation and tumorigenesis (represented with black arrows). Adapted from [21,23].

Medulloblastoma (MB) is the most frequent pediatric solid tumor, representing around 20% of the tumors of the CNS in childhood [17]. This tumor arises in the cerebellum and it is classified as a grade IV lesion by the World Health Organization (WHO) [18]. As mentioned above, cerebellum development needs a well-regulated rate of proliferation and differentiation of the CGNPs in order to form a correct cerebellum [19]. The SHH–Patched (PTCH1) [20], or WNT [21] signaling pathways are key regulators of this process. PTCH1 is the receptor of SHH. In the absence of SHH, PTCH1 inhibits the Smoothened (SMO)-GLI signaling pathway (Figure 2). When SHH is present, its binding to PTCH1 releases the negative regulation that PTCH1 is exerting to SMO and therefore the signaling pathway will be active, promoting cell proliferation [20]. The mechanism of action of the WNT signaling pathway is similar. As represented in Figure 2, in the absence of WNT, the multiprotein complex formed by Axin, and the Glycogen Synthase Kinase 3 Beta (GSK3β) and APC will phosphorylate cytoplasmic β-catenin that will then be degraded. When WNT is present, it will bind to its receptor Frizzled and this binding will inhibit the GSK3β inhibiting the function of the multiprotein complex. β-catenin will be accumulated and then it will translocate into the nucleus, promoting the cell cycle progression. If an excessive activation of these signaling pathways occurs, the MB progression may occur because of the incapacity of the cells to exit from a proliferative state and enter in a differentiation process [22]. Indeed, the patients with Gorlin’s syndrome (PTCH1 mutation) and the patients with Turcot’s syndrome (APC mutation) present increased incidence of MB [20]. Further evidence demonstrating that the hyperactivation of these signaling pathways are responsible for the loss of equilibrium in cerebellar growth are the WNT and SHH MB mice models [23,24,25].

The progress in MB therapy has increased the survival rate of the patients, although it is very variable depending on the tumor subtype. The strategy that is used nowadays for MB patients is based on maximal tumor resection, followed by chemotherapy and craniospinal radiotherapy [17]. The prognostic of the patients is different depending on the type of resection (complete or not) and the age of the patient, since patients younger than 3 years old cannot receive craniospinal radiotherapy because of the risks that promote a second neoplasia [26]. Regarding to chemotherapy, the most used strategy is the combination of lomustine, vincristine, and cisplatin [17]. This therapy has improved the survival of patients, in some cases reaching a 70–90% of survival rate [27]. However, the high doses of chemotherapy and radiotherapy that need to be used in order to achieve a therapeutic response cause irreparable damage to the healthy tissue, causing permanent neurological sequelae and disability [28,29]. Furthermore, despite the improvement in the prognosis for children with MB, about 30% of the surviving patients relapse after the initial treatment [29,30].

Moreover, almost 30% of the patients present with disseminated tumor at the moment of diagnosis [31]. MBs spread from the cerebrospinal fluid (CFS), and the most common dissemination is leptomningeal. Metastases outside the CFS are very rare, but they can appear in the bone, lymph node, and lung, in decreasing order of occurrence [32].

## 4. Medulloblastoma Subgroups

The first classification of MBs was based on their histopathological features. Until 2007, three main subgroups were defined (classic, desmoplastic-nodular, and large cell anaplastic [LCA] MB), when the WHO classification defined four different histological subgroups, namely, desmoplastic-nodular, large cell, anaplastic, and MB with extensive nodularity (MBEN), the first one being the one with the best prognosis [33]. This classification was supplemented in 2011 with the studies of Northcott and colleagues. Using a bioinformatic analysis of transcriptional data from two cohorts from Toronto and Moscow, they discovered four distinct molecular variants of MB, which they denominated WNT, SHH, Group C, and Group D [34]. These and additional studies gave rise to a consensus conference in Boston in 2010, where the discussants came to a consensus of the existence of four MB subgroups, named WNT, SHH, Group 3, and Group 4 [35]. In a recent study that was carried out by Cavalli and colleagues, 763 MB samples were analyzed using the similarity network fusion approach [36]. The result of this work was the identification of new subtypes within the four MB subgroups, specifically, they identified a total of 12 subtypes, namely: two WNT, four SHH, three Group 3, and three Group 4 subtypes [36]. The main features and the relationship among these classifications are summarized in Table 1.

### 4.1. WNT subgroup

This subgroup is characterized by the aberrant activation of the WNT/β-catenin signaling pathway and the good prognosis of the patients [37]. In 2017, two subtypes were identified, WNT α and WNT β. The main molecular difference between the two subgroups is that the WNT α type tumors present monosomy 6, where β-catenin gene is located, whereas the WNT β type tumors are normally diploid for chromosome 6 (Table 1). The WNT α tumors are frequent in children and adolescents, whereas the WNT β tumors are more likely to appear in adolescents and adults [36] (Table 1).

Table representing key histological and clinical data of two different MB classifications, as well as genetic alterations, metastasis rates, age, and survival data of the Cavalli classification. The mTOR implication in some of the subgroups is also presented. The age groups are infant (0–3 years), children (>3–10 years), adolescent (>10–17 years), and adult (>17 years). Ado—adolescent; amp: amplification; child—children; dup—duplication; LCA—large cell/anaplastic; MBEN—medulloblastoma with extensive nodularity; mut—mutations; ↑—activation.

### 4.2. SHH Subgroup

This subgroup is characterized by the aberrant activation of the SHH signaling pathway that gives rise to the disease. Different genes belonging to this signaling pathway can be mutated, such as SHH, PTCH, SMO, SUFU, GLI1, or GLI2. The patients that are classified in the SHH subgroup have an intermediate prognosis, except the infants who have a good prognosis. This information has recently been complemented with the four subtypes that are defined within the SHH group: SHH α, SHH β, SHH γ, and SHH δ [36] (Table 1). The SHH α subtype mainly affects the children and adolescents, and, among other alterations, it is the only one presenting the TP53 mutations [36]. Furthermore, this subgroup is enriched with the expression of the genes that are involved in DNA repair and cell cycle progression [36]. Both SHH β and SHH γ tumors affect infants, but the survival rates are very different. As shown in Table 1, the SHH β tumors are characterized by phosphatase and tensin homolog (PTEN) loss and they present the lowest survival rate at 5 years within the SHH tumors. In addition to identifying specific mutations and genetic alterations, Cavalli and colleagues identified an enrichment on the developmental signaling pathways in the SHH β and SHH γ subgroups. In SHH δ, the main characteristic is the enrichment of mutations in TERT promoter [36] (Table 1). 

### 4.3. Group 3

This subgroup presents the worst survival rate [38] and to date, a unique altered signaling pathway originating the disease has not been identified. Cavalli and colleagues have described three different subtypes within the Group 3 MB, namely: Group 3α, Group 3β, and Group 3γ. Group 3α is characterized by chromosome 8q loss (encoding v-myc avian myelocytomatosis viral oncogene homolog (MYC)). The Group 3β tumors are characterized by the activation of the GFI1 and GFI1B oncogenes, amplification of OTX2, and loss of DDX31 on chromosome 9. Finally, the Group 3γ tumors present the MYC amplification as a result of the gain of chromosome 8q [36] (Table 1).

Regarding to the implication of the signaling pathways, the Group 3α tumors are enriched in the expression of the photoreceptor, muscle contraction, and primary cilium-related genes, while Group 3β and 3γ present the enrichment of protein translation pathways. Furthermore, Group 3γ is also enriched in the expression of genes that are related to telomere maintenance [36].

### 4.4. Group 4

This is the most prevalent group; almost 40% of the MBs are included in this subgroup. As in Group 3, the deleterious signaling pathway that causes the disease has not been identified. There are three different subtypes that have been described within this subgroup: Group 4α, Group 4β, and Group 4γ. The main characteristics of the Group 4α tumors are the v-myc avian myelocytomatosis viral-related oncogene, neuroblastoma-derived (MYCN) and cyclin dependent kinase 6 (CDK6) amplifications, 8p loss and 7q gain. The Group 4β tumors are characterized with the synuclein alpha interacting protein (SNCAIP) duplications and ubiquitous i17q. Finally, the Group 4γ tumors present CDK6 amplifications, 8p loss, and 7q gain, as Group 4α tumors, but with the absence of MYCN amplifications [36] (Table 1). 

The experiments that were performed by Cavalli and colleagues have identified differentially activated pathways for each subtype, supporting the existence of the three independent subtypes. The pathways that were identified were the activation of migration pathways in Group 4α, activation of mitogen activated kinase-like protein (MAPK) and fibroblast growth factor receptor 1 (FGFR1) signaling pathways in Group 4β, and activation of PI3K/AKT/mTOR and erb-b2 receptor tyrosine kinase 4 (ERBB4)-mediated nuclear signaling pathways in Group 4γ [36].

It is of relevance to underscore that in 2010, Gibson and colleagues demonstrated that the distinct subgroups of MB have different developmental origins, discovering that some WNT MBs were arisen from the cells in the dorsal brainstem [23]. Later, Grammel and colleagues discovered that some of the SHH MBs arise from the granule neuron precursors of the cochlear nuclei of the brainstem [39]. Two independent studies also reported two different cells of origin for Group 3 MB, with stem cell characteristics [40,41]. At last, several cell types were shown to be able to give rise the Group 4 tumors [26]. What all of these cells of origin have in common are the stem cell properties, and it is relevant to take into account that the majority of MB cells have a stem-like appearance [26].

## 5. Medulloblastoma Stem Cells (MBSCs)

The cancer stem cell (CSC) hypothesis explains the existence of a small fraction of tumor cells that have stem properties and the ability to proliferate and maintain the tumor growth [42]. These cells are characterized by two main properties, self-renewal and differentiation capacity, the self-renewal being the key property regulating the oncogenic potential, so that tumorigenesis is an effect that is derived by a deregulation of this process [43]. In the last years the presence of CSC has been described in different hematopoietic and solid-tumors, including MB [44]. These MBSCs are characterized by high levels of expression of CD133, Sox2, Musashi1, and Bmi1, which are all neural stem cell (NSC) genes [45]. As it occurs with CSCs, the MBSCs are considered responsible for therapeutic resistance and tumor recurrence [26], which is common in these tumors. Therefore, it would be necessary to develop targeted therapies against these MBSCs, in order to avoid tumor resistance and recurrence. Different studies have been focused on targeting different signaling pathways that are implicated in MBSCs, such as the SHH, PI3K/AKT/mTOR, Stat3, and Notch signaling pathways [26]. 

## 6. mTOR in Cancer

The activation of the mTOR pathway plays a key role in the development of several cancer types because of its importance in controlling cell growth and metabolism [1]. Aberrant mTOR activation can occur through oncogene stimulation or the loss of tumor suppressors [46]. Although the constitutive activation of the mTOR gene can occur, mutations in downstream and upstream components of both mTORC1 and mTORC2 [46] are more frequent, and these mutations are responsible for inducing cancer cell growth, survival, and proliferation [1]. 

The PI3K/AKT signaling pathway is found to be deregulated through a variety of mechanisms in many human cancers [1]. Mutations in different components produce constitutive activation of this signaling pathway, leading to a disturbance between the cell proliferation and apoptosis [47]. For instance, PIK3CA amplifications/mutations, AKT overexpression, and PTEN loss have been described in breast [48] or colorectal cancer [49].

Downstream of mTORC1, the overexpression of S6K1, 4EBP1, and eIF4E has been associated to cellular transformation [46]. eIF4E overexpression occurs in different human tumors, like breast, head and neck, colon, prostate, bladder, cervix, and lung cancer, enabling the selectable translation of some mRNAs that encode key proteins for cellular transformation [50]. Additionally, the loss of p53, a common event in cancer [1], negatively regulates some of the downstream targets of mTORC1, such as autophagy [51]. Thus, the function of the mTOR pathway in cancer development makes it interesting for targeted therapy in different tumors.

## 7. mTOR in Medulloblastoma

During brain development, the mTOR-mediated signaling pathway masters the differentiation of neurons and glia, as well as the maintenance of the stemness of NSCs [14]. In the expansion phase of CGNPs in the cerebellum, SHH and IGF are required [16], and it has been suggested that the activation of both pathways in CGNPs could interact and enhance tumor formation in the cerebellum [52,53,54]. Following this hypothesis, Rao and colleagues discovered that, in mice, the SHH induced tumor formation increases significantly when IGF-II is co-expresed, but no tumor formation was observed in the mice that were injected with IGF-II alone [53]. 

IGF positively regulates the mTOR pathway, which is frequently activated in malignant brain tumors, including MB [55]. Such an activation promotes the upregulation of protein translation by inhibition of 4E-BP1, through mTORC1 mediated phosphorylation [56]. Besides, a growing body of evidence points to the SHH signaling pathway as being the responsible for promoting the activity of mTORC1/4E-BP1-dependent translation and enhance tumor formation [6,57]. This evidence suggests that IGF-II, and therefore, the mTORC1/4E-BP1 pathway is a downstream transcriptional target of SHH, being critical in the SHH-mediated MB [58]. 

As a result of its role in the canonical SHH signaling pathway, mTORC1 seems to be a potentially important molecular target for treating SHH MBs [57]. However, the SHH signaling pathway also interacts with additional signaling pathways to promote MB growth and to induce treatment resistance. For instance, the Hippo pathway plays an important role in the control of organ development, and cross-talks with this pathway have been described [59].

Regarding the role of the PI3K/AKT/mTOR signaling pathway, its activation occurs in subgroup 4γ, the most prevalent subtype [36]. Moreover, Frasson et al. reported that the PI3K inhibition induces dramatic morphological changes and promotes apoptosis in DAOY human MB cells [60]. All of these data together suggest that targeting mTOR could be a potential therapeutic strategy for SHH-driven and Group 4 MBs. However, no relation between mTOR and the WNT or Group 3 MB subgroups has been described yet.

## 8. mTOR Signaling Pathway in MBSCs

Like all CSCs, the MBSCs possess the ability of self-renewal and differentiation, increasing the oncogenic potential of the heterogeneous tumor [61]. The transcription factors octamer-binding transcription factor 4 (OCT4), Nanog homeobox (NANOG), and SRY-box 2 (SOX2) are essential to maintain the pluripotency and self-renewal in embryonic stem cells and CSCs [14,62]. In addition to the expression of these transcription factors, one of the leading pathways that are involved in the regulation of embryonic stem cell differentiation and resistance of CSCs to therapy is the PI3K/AKT/mTOR signaling pathway [60]. This signaling pathway plays an essential role in the maintenance and survival of the CSCs by regulating multiple apoptosis-related proteins [61] and controlling the cell cycle progression [55]. Therefore, mTOR-mediated intracellular signaling is tightly regulated in the stem cells and it is considered one of the key modulators of the stemness in different stem cell populations [62]. Besides, it has been demonstrated that when inhibiting the mTOR signaling pathway, the CSC properties are reduced and the invasion potential is restrained in some of the cancer types [63].

The fact that PI3K inhibition has a heavy impact on the cell number of primary MB cells has already been demonstrated [60]. Following the hypothesis that stem cells could be the preferential target of PI3K/AKT inhibition, Frasson and colleagues showed that such inhibition indeed targets the CD133 positive cell fraction, reducing the number of the MBSC pool [60]. Furthermore, Hambardzumyan and colleagues discovered that the PI3K⁄AKT inhibitor, perifosine, increases the sensitivity to radiation-induced apoptosis in Nestin positive MBSCs [64]. Additionally, the PI3K/AKT axis is able to enhance the intracellular SHH signaling in CGNPs [65]. Different studies support the idea that the SHH signaling is also important in the regulation of CSC [66,67]. Ahlfeld and colleagues described that the constitutive activation of SHH signaling results in a significantly augmented expression of Sox2 that induces the cellular growth and proliferation of SHH MBs [68]. Therefore, targeting CSCs by the inhibition of mTOR or SHH may improve the outcome of patients with MB.

## 9. Targeted Therapy

As mentioned above, the current therapy is not enough to cure all of the MB patients, and the high doses of chemotherapy and radiotherapy that are needed induce severe side effects. This is the reason that recent investigations are directed toward improving targeted therapy in MB subgroups. There are two main objectives; on the one hand, to discover new drugs, and on the other hand, to optimize the doses of the drugs that are usually used. One consideration to be taken into account in MB treatment, as it occurs in all brain tumors, is that the drug must be able to cross the blood-brain barrier, which makes the development of new therapeutic agents difficult. 

### 9.1. Targeting WNT Medulloblastomas

The WNT MBs present a good rate of cure compared with the other subgroups, since the WNT activation increases the tumor’s radiosensitivity [69]. That is the reason that there are relatively few drugs that have been developed to target this signaling pathway. The current clinical trials are focused on the refinement of the standard treatment, with the objective of reducing the doses of chemotherapy and radiotherapy to decrease the neurotoxic side effects that are related to the treatment [27]. 

Two specific therapies have been developed to target the WNT MBs. The first one, norcantharidin, has been shown to block the WNT signaling pathway, impairing the growth of the MB [70]. The second one, lithium chloride, inhibits the GSK3β stabilizing β-catenin and reduces the MB growth [71].

### 9.2. Targeting SHH Medulloblastomas

Many specific treatments for SHH MB have been developed. Almost all of these treatments are focused on inhibiting SMO, a G protein type receptor that is implicated in the SHH signaling pathway. These treatments are based on the structure of cyclopamine, a naturally occurring plant alkaloid, the first SHH pathway inhibitor that was discovered with an anticancer effect. Cyclopamine inhibits the SMO protein, binding to its transmembrane domain and avoiding its change of conformation to the active form. However, cyclopamine has failed in clinical development, mainly because of its pharmacokinetic characteristics [26]. Therefore, research is focused on the development of new small molecules based on this compound, but with improved pharmacokinetic properties. Several small molecules have been developed, like vismodegib (GDC-0449), saridegib (IPI-926), erismodegib (LDE-225), TAK-441, XL-139 (BMS-833923), PF-04449913, and PF-5274857 [26].

The most studied of all of these analogs is vismodegib, the first food and drug administration (FDA)-approved drug as a SHH signaling inhibitor for advanced and metastatic basal cell carcinoma [72]. The patients with SHH-driven MB that were treated with vismodegib had a remarkable response and tumor size regression [73]. However, as vismodegib is a SMO inhibitor, it is not an effective treatment for the patients harboring genetic aberrations in genes downstream SMO, such as SUFU or GLI2 [74]. Furthermore, a number of patients that were treated with vismodegib acquired drug resistance because of a point mutation in SMO, the D473H mutation. This mutation would not prevent the activation of the SHH signaling pathway, but it would disrupt the ability of vismodegib to bind to the SMO [75]. Thus, a therapy targeting GLI using bromo- and extra-terminal BET domain inhibitors may be an alternative and efficient treatment for patients with genetic aberrations in SUFU or GLI, as well as for the patients who acquire resistance to SMO inhibitors, since they modulate GLI expression downstream of SMO and SUFU [76]. Another method to target GLI is the use of arsenic compounds. They have been tested in vitro and in vivo as a treatment for SHH-driven cancers and they have showed promising results [77].

### 9.3. Targeting Group 3 and 4 Medulloblastomas

The lack of an altered signaling pathway responsible for initiating the tumor makes it difficult to develop a targeted therapy for these subgroups of MBs. Among other alterations, some Group 3 MBs are characterized with MYC overexpression [36,74]. Two FDA-approved drugs targeting MYC, pemetrexed and gemcitabine, were used in combination to treat mouse allografts and xenografts. As a result of the treatment, the tumor growth was decreased [78]. Additionally, BET bromodomaim proteins have also been demonstrated to inhibit MYC-regulated signaling pathways in different cancers. Therefore, targeting these proteins could also be a promising strategy to target this subgroup of MBs. Nevertheless, MYC overexpression is only found in 10–20% of the patients in subgroup 3 [79].

### 9.4. Targeting the mTOR Pathway

As a result of the importance of the PI3K/AKT/mTOR pathway in cancer progression, targeting this signaling pathway has become one of the most studied strategies. Rapamycin is the first mTOR inhibitor that has been used in anti-cancer therapy. This compound is an antifungal agent that binds to FK506 Binding Protein 12 (FKBP12), forming a complex that allosterically inhibits the FKB12-Rapamycin Binding (FRB) domain of mTORC1, leading to the dissociation of Raptor from mTORC1 [80]. As rapamycin inhibits the mTORC1 complex, it also inhibits the protein translation and synthesis, and it induces cell cycle arrest in the G1 phase [10]. Even if rapamycin is not able to inhibit mTORC2, it can affect the mTORC2 complex indirectly [10,55]. However, poor solubility and unpredictable pharmacokinetic profiles of rapamycin have led to the development of rapamycin derivatives (rapalogs), new compounds based on the structure of rapamycin, but with improved pharmacological properties. These compounds have been tested and are approved for use in the treatment of different solid tumors [81,82,83,84,85,86,87,88,89,90,91,92,93,94,95,96,97,98,99] (Table 2).

Some of these rapalogs have also been tested in MB, demonstrating promising results and leading to three clinical trials in phase I. These compounds are temsirolimus, sirolimus, and tesirolimus, in combination with perifpsine, an AKT inhibitor [55]. 

The PI3K/AKT/mTOR signaling pathway is primarily involved in SHH MBs. In these types of tumors, not only the mutations in the SMO receptor or the aberrant activation of the SHH pathway are the cause of MB initiation; an increased activation of the PI3K signaling or the alternative activation of the RAS-MAPK pathway could also eventually cause drug resistance [100]. Thus, combining SMO and PI3K/AKT/mTOR inhibitors may be a strategy to overcome resistance development.

**Figure 3:**
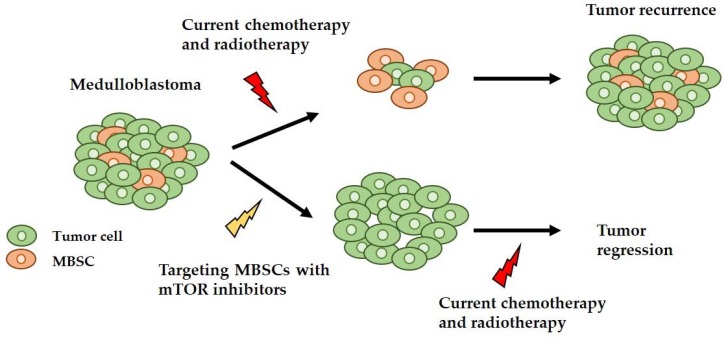
Schematic representation of a possible new approach to target medulloblastoma stem cells (MBSCs)s and MB. Medulloblastoma has an intracellular heterogeneity, having different cell types, such as normal tumor cells and MBSCs. With the classic treatment, the elimination of normal tumor cells is achieved, and the surviving MBSCs can form the tumor again. With the proposed new approach, the MBSCs are eliminated using mTOR inhibitors and the tumor cells are eliminated using the conventional therapy, achieving total tumor regression.

Finally, it has been shown that the PI3K/AKT/mTOR signaling pathway is implicated in CSCs, and specifically in MBSCs, demonstrating that the inhibition of this signaling pathway reduces the MBSC population in the primary tumor culture [60]. Therefore, inhibiting mTOR may be a potential treatment to target the MBSCs, thus reducing the chances of tumor recurrence and therapy resistance (Figure 3).

## 10. Concluding Remarks 

MB is the most common malignant solid tumor in childhood, and even if the current therapies have improved the overall survival, the side effects that they generate are devastating for children. Improved knowledge of MB has given rise to a new and detailed classification of the subgroups of MB, which may lead, in the future, to a better stratification of the patients, based on the molecular characteristics of their tumor, moving towards a personalized therapy for each patient. To reach this goal, a deeper molecular profiling of each tumor is needed after the biopsy or surgery. Together with the new classification, new signaling pathways that are implicated in different subgroups of MB, have been identified. Signaling pathways such as the SHH or WNT signaling pathways, as well as PI3K/AKT/mTOR are therapeutic targets in MB.

mTOR is a master signaling pathway that regulates organismal growth and homeostasis, as a result of its implication in protein and lipid synthesis, and in the control of the cell cycle and the cellular metabolism. Different studies have shown that it is also necessary in cerebellar development and stem cell pluripotency maintenance. Being an essential protein in the homeostasis of the cells, when it is deregulated, it is implicated in different tumors, including MB. Furthermore, it has a decisive role in MBSCs, which is demonstrated by the fact that when the PI3K/AKT/mTOR signaling pathway is inhibited, the number of MBSCs decreases. 

In this review we describe the targeting of mTOR as a promising therapeutic approach, mainly, but not only, for SHH-driven MB patients. Moreover, the combination of this approach with the current therapies could be a promising strategy, as the mTOR inhibition impairs the growth of MBSCs and increases the sensitivity to radiation-induced apoptosis in Nestin positive MBSCs, decreasing the possibility of tumor recurrence and therapy resistance (Figure 3).

## Figures and Tables

**Table 1:** Graphical summary of the different classification of medulloblastoma (MB) subgroups and their specifications.

Medulloblastoma Classification System	Clinical Features	WNT		SHH				Group 3			Group 4		
**Taylor Classification**	**Histology**	Classic, Rarely LCA		Desmoplastic/Nodular, Classic, LCA				Classic, LCA			Classic, LCA		
	**Prognosis**	Very good		Infants Good; Other Intermidiate				Poor			Intermidiate		
**Cavalli classification**		**α**	**β**	**α**	**β**	**γ**	**δ**	**α**	**β**	**γ**	**α**	**β**	**γ**
	**Metastasis**	9%	21%	20%	33%	9%	9%	43%	20%	40%	40%	41%	39%
	**Genetic alterations**	Monosomy 6		TP53 mutations	PTEN loss		TERT promoter mut	8q loss	GFI1 and GFI1B ↑, OTX2 amp, DDX31 loss	MYC amp	MYCN and CDK amp, 8p loss, 7q gain	SNCAIP dup, i17q	CDK amp, 8p loss, 7q gain
	**Age**	Child; Ado	Ado; Adult	Child; Ado	Infant	Infant	Adult	Infant; Child	Child; Ado	Infant; Children	Child; Ado	Child; Ado	Child; Ado
	**Subtype histoloogy**			LCA, desmoplastic	Desmoplastic	MBEN, desmoplastic	Desmoplastic						
	**Survival**	97%	100%	70%	67%	88%	89%	66%	56%	42%	67%	75%	83%
**mTOR**				mTORC1 activation									PI3K/AKT/mTOR activation

**Table 2:** Mechanistic target of rapamycin (mTOR) inhibitors used in the clinic.

Types of mTOR Inhibitors	Name	Target	Disease	Trial Phase
Rapalogs	Temsirolimus	mTOR	RCC and MCL	Completed phase III
	Everolimus	mTOR	RCC, PNET, Lung, GEP, NET, Gastric, BC, mRCC	Completed phase III
	Ridaforolimus	mTOR	Sarcoma	Completed phase III
Second-generation mTOR inhibitors	BEZ235	PI3K/mTOR	BC, RCC, Endometrial, PNET	Discontinued
	GSK2126458	PI3K/mTOR	Colon/Rectum, RCC, BC, Endometrial, Melanoma, Ovary/Primary Peritoneal, Pancreas, Prostate	Phase I
	Gedatolisib (PF-04691502; PKI-587)	PI3K/mTOR	SCLC, Ovarian, Endometrial, Renal, Colorectal, Glioblastoma	Phase I
	Apitolisib (GDC-0980)	PI3K/mTOR	MPM, Colorectal, GIST, Sarcoma, BC	Phase I
Third-generation mTOR inhibitors	Rapalink-1	mTOR (mutant forms too)	Glioblastoma	No clinical data

mRRC—metastatic renal cell carcinoma; MCL—mantle cell lymphoma; PNET—pancreatic neuroendocrine tumor; GEP—gastro-entero-pancreatic; NET—neuroendocrine tumor; BC—breast cancer; SCLC—small cell lung cancer; MPM—malignant, pleural mesothelioma; GIST—gastrointestinal stromal tumor.
